# Supplementary material for: Sustained-input switches for transcription factors and microRNAs are central building blocks of eukaryotic gene circuits
Source: Genome Biol. 2013 Aug 23;14(8):R85. doi: 10.1186/gb-2013-14-8-r85 (PMC4054853; doi:10.1186/gb-2013-14-8-r85)
Supplement: Additional file 5 — HTML Browsable Motif Output. Zipped folder containing all WaRSwap and FANMOD motif output, viewable in a web browser. [file gb-2013-14-8-r85-S5.ZIP › HTML_browsable_motif_output/WaRSwap_ath_tair10/sigs_WSWRSLDraw_TAIR10-2500.pvals.heatmaps.html/motif_id_46_000101110_tftype_ath_upstream_-3000_0.html]

```
BG_MODEL = WARSwap
MOTIF_ID = 46_000101110
TF_TYPE = ath
UPSTREAM = -3000_0


PVals
FNR = 0.2	FNR = 0.4	FNR = 0.6	FNR = 0.8
deltaG = 60	0.242	0.001	0	0.722
deltaG = 70	0.237	0.003	0	0.697
deltaG = 80	0.254	0.003	0	0.698

ZScores
FNR = 0.2	FNR = 0.4	FNR = 0.6	FNR = 0.8
deltaG = 60	0.724	2.349	3.222	-1.012
deltaG = 70	0.714	2.308	3.201	-0.957
deltaG = 80	0.688	2.288	3.172	-0.967

StDevs
FNR = 0.2	FNR = 0.4	FNR = 0.6	FNR = 0.8
deltaG = 60	138.529	69.148	29.42	2.387
deltaG = 70	134.724	71.106	29.752	2.437
deltaG = 80	141.56	70.741	29.996	2.468
```
